# Bronchial or Laryngeal Obstruction Induced by Exercise?

**DOI:** 10.3389/fped.2017.00150

**Published:** 2017-06-28

**Authors:** Ayoub Bey, Sophie Botti, Laurianne Coutier-Marie, Claude Bonabel, Stéphanie Metche, Silvia Demoulin-Alexikova, Cyril Etienne Schweitzer, François Marchal, Laurent Coffinet, Iulia Ioan

**Affiliations:** ^1^Service d’ORL pédiatrique, Hôpital d’enfants, CHRU de Nancy, Vandoeuvre, France; ^2^Service d’explorations fonctionnelles pédiatriques, Hôpital d’enfants, CHRU de Nancy, Vandoeuvre, France; ^3^DevAH EA 3450, Faculté de Médecine, Université de Lorraine, Vandoeuvre, France; ^4^Service de Pédiatrie, Hôpital d’enfants, CHRU de Nancy, Vandoeuvre, France

**Keywords:** asthma, respiratory resistance, spirometry, dyspnea on exertion, forced oscillations

## Abstract

A child suspected of exercise-induced laryngeal obstruction and asthma is examined by laryngoscopy and respiratory resistance (Rrs) after exercise challenge. Immediately at exercise cessation, the visualized adduction of the larynx in inspiration is reflected in a paroxystic increase in Rrs. While normal breathing has apparently resumed later on during recovery from exercise, the pattern of Rrs in inspiration is observed to reoccur following a deep breath or swallowing. The procedure may thus help diagnosing the site of exercise-induced obstruction when laryngoscopy is not available and identify re-inducers of laryngeal dysfunction.

Exercise-induced laryngeal obstruction (EILO) presents with paroxysmal dyspnea, labored, noisy breathing and may be misdiagnosed for asthma, namely, with exercise-induced bronchoconstriction. The coexistence of EILO and asthma may be a further complication to the diagnostic (1). Laryngoscopy, especially throughout an exercise test has been described as an optimal procedure (2), but alternative methodology may be helpful whenever an ENT specialist is not readily available in the ward, or the child is reluctant to undergo a somewhat more invasive procedure than lung function testing. The respiratory resistance (Rrs) is measured non-invasively by the forced oscillation technique and may conveniently be used to describe the time course of airway caliber in relation with breathing, especially during prolonged observation. Acute patterns have been described in children where the exercise-induced paradoxical vocal cord motion had otherwise been authenticated by laryngoscopy (3, 4). In this paper, we present a coordinated characterization of exercise-induced paroxysmal dyspnea. The Rrs dynamics and its confrontation to spirometry allowed identifying findings that characterize the respective contribution of EILO and asthma, as well as re-inducers of the laryngeal obstruction once the exercise has been completed.

A 12-year-old boy exhibited several episodes of acute dyspnea triggered by exercise for about 2 years, in a context of chronic cough and occasional night time dyspnea. One such acute episode required admission to the emergency room, where the paradoxical vocal cord adduction in inspiration was evidenced by laryngoscopy.

Several weeks after the threatening episode, the child returned to hospital and underwent an exercise-challenge protocol including videolaryngoscopy and lung function measurements, Rrs and spirometry, so as to assess the respective contribution of EILO and asthma. Spirometry measured the forced vital capacity (FVC), the forced expiratory volume in 1 s (FEV1), and the FEV1/FVC ratio as previously described (5). In this protocol, after a few tidal breaths, the child was instructed to inspire maximally from the functional residual capacity and expire forcefully to residual volume. The Rrs was measured by the forced oscillation technique that provides 12 Rrs measurements per second and has therefore the resolution necessary to estimate the within-breath change in airway caliber (6). The current signal analysis includes a filtering algorithm to eliminate those measurements corrupted by major flow distortion (7). Rrs acquisition lasts approximately 1 min during which the child was asked to take a deep breath (8), a routine maneuver that is implemented in our protocol to test bronchial tone reversibility (9).

The submaximal exercise test consists in an 8 min run on a treadmill in a climate room where ambient air absolute humidity is maintained below 10 mg/L (10). The videolaryngoscopy was performed by the pediatric ENT specialist. A 2.5 mm × 270 mm STORZ REF 11101SK2 videolaryngoscope was used without xylocaine nebulization and lasted approximately 2 min. Lung function measurements, Rrs and spirometry in that order were followed by videolaryngoscopy at baseline. Videolaryngoscopy was repeated immediately at cessation of exercise. Rrs and spirometry were obtained again in that order 5 and 15 min after exercise, as well as 20 min following inhalation of 400 µg salbutamol.

Written informed consent was obtained from the child and his parents to participate in this study and to publish his case report.

The child was heard to cough on several occasions while performing forced expiration maneuvers as well as when exercising, with labored breathing and inspiratory dyspnea occurring toward the end of the run.

The videolaryngoscopy identified a normal vocal cord pattern of adduction in expiration at baseline while, immediately after exercise, a biphasic laryngeal dysfunction was demonstrated. The fast onset, slow resolution inspiratory narrowing involved both supraglottic (arytenoid cartilage) and glottic (true vocal fold) levels. The paradoxical vocal cord adduction during inspiration and abduction during expiration are illustrated in Figure 1.

**Figure 1:**
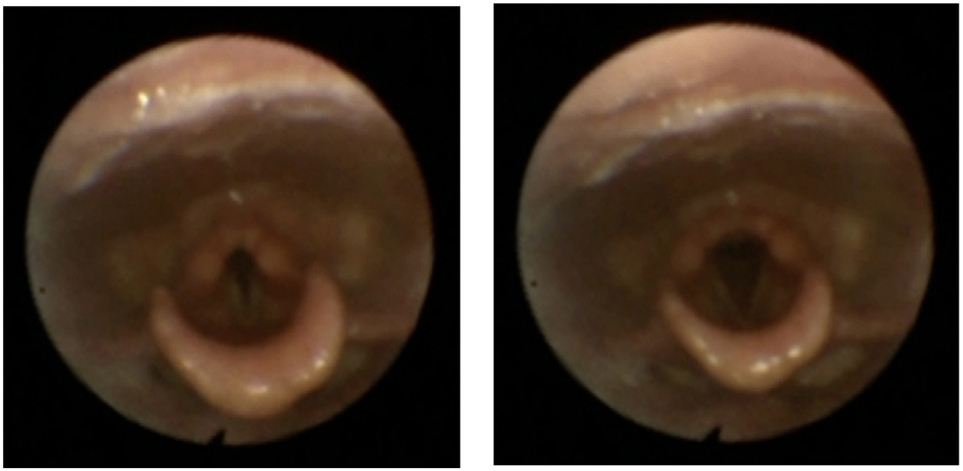
Pictures from the videolaryngoscopy taken immediately after exercise showing the vocal cord adduction in inspiration (left) and abduction in expiration (right).

The time course of Rrs at baseline showed the expected slight flow-dependent increase during both inspiration and expiration (11) (Figure 2). A few minutes after exercise, while less labored and somewhat more regular breathing had resumed, Rrs showed first a normal pattern for a few breaths, but when airflow was briefly interrupted in expiration—probably with swallowing—a sudden, large increase in Rrs occurred in inspiration (Figure 3), a pattern that continued throughout the acquisition, especially not resolving with the deep breath. The assessment 15 min after exercise also showed at first a normal pattern, but the deep inhalation was associated with major and irregular increase in Rrs in inspiration (Figure 4). There was no significant variation in Rrs due to occasional re-occurrence of laryngeal obstruction after bronchodilator inhalation.

**Figure 2:**
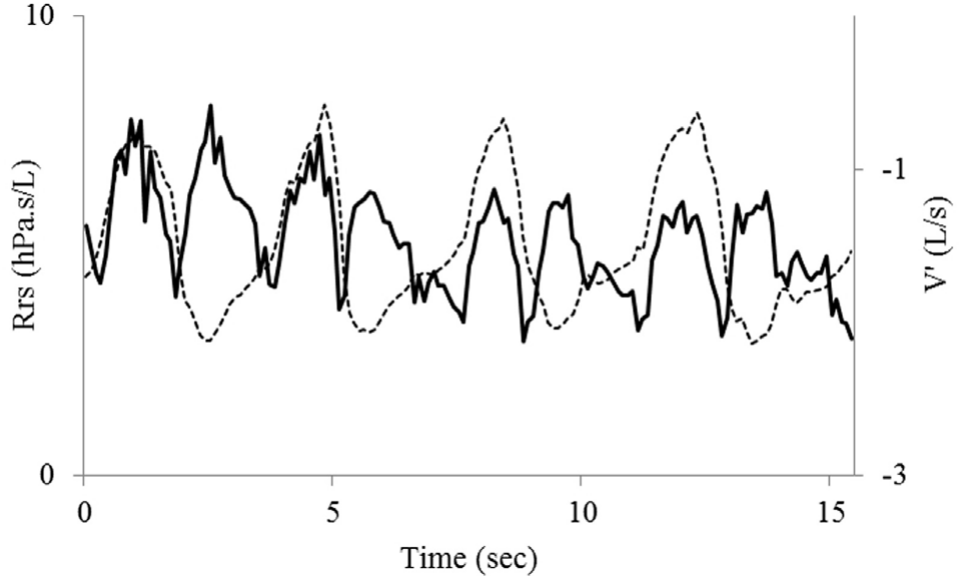
Baseline recording of tidal flow (V′, dotted line)—inspiration upward—and respiratory resistance (Rrs, bold line). The tracings are superimposed to emphasize the normal Rrs variation that follows the absolute value of flow in both inspiration and expiration.

**Figure 3:**
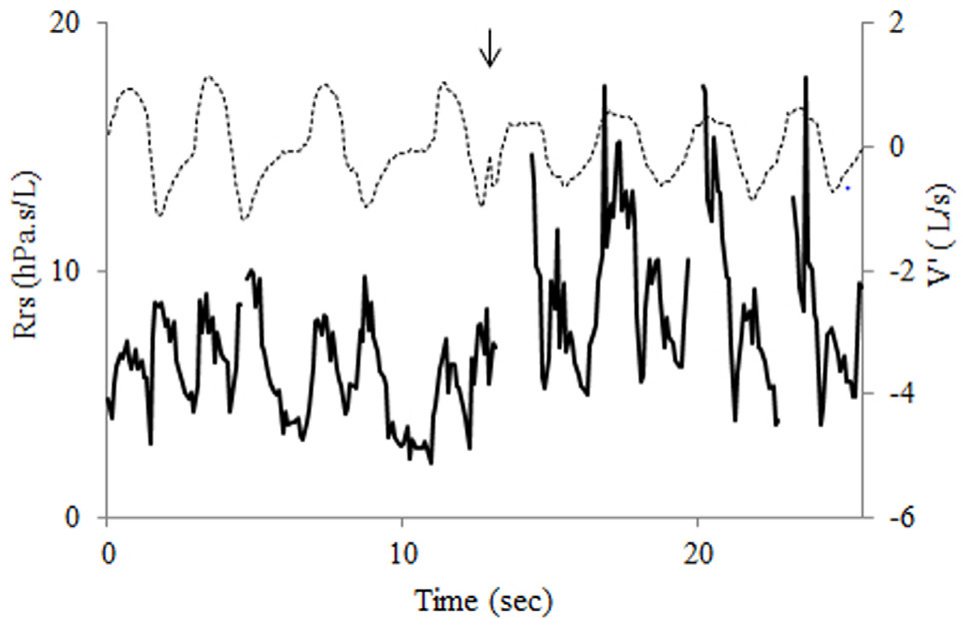
Recording of flow (V′, dotted line) and respiratory resistance (Rrs, bold line) 5 min after exercise shows first a normal Rrs pattern for three breaths. The transient interruption of expiratory flow at fourth breath (arrow) is followed by a dramatic increase in Rrs during the following inspiration that also shows flow limitation. The pattern is continued for the next inspirations. Note transient interruptions of Rrs trace due to the filtering procedure.

**Figure 4:**
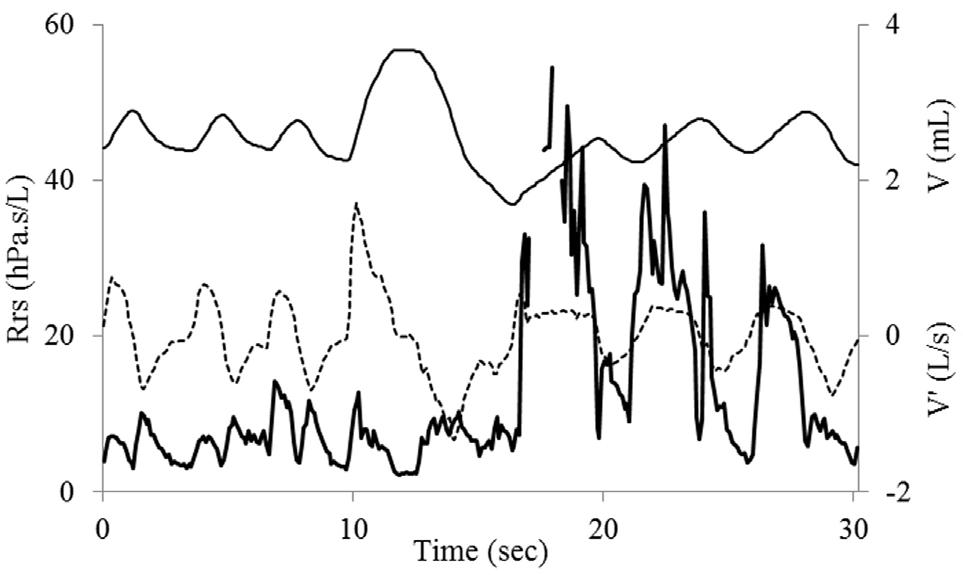
Recording of flow (V′, dotted line), tidal volume (V, light), and respiratory resistance (Rrs, bold line) 15 min after exercise. The normal Rrs pattern is dramatically altered after the deep breath. A large increase in Rrs in inspiration occurs during the next few breaths. Also note inspiratory flow limitation on first breath after deep inhalation.

At baseline FVC and FEV1 *z*-scores were, respectively, −0.40 and −1.12 and the FEV1 to FVC ratio was 77%, i.e., close to the predicted lower limit of normal (12). Both FEV1 and FVC showed a 7% reduction from baseline 5 min after exercise but were, respectively, 7 and 8% *higher* after 15 min, with no change in FEV1/FVC at either time. A pattern of inspiratory flow limitation was occasionally observed during spirometry maneuvers 5 min after challenge but could not be quantitated with the current protocol. After bronchodilator inhalation, FEV1 and FVC increased, respectively, 19 and 10% from baseline, and the FEV1/FVC ratio was up to 84%.

The clinical course of EILO was acutely induced during a short exercise, the laryngeal adduction visualized within seconds of the run, and major alterations of Rrs observed a few minutes thereafter. The videolaryngoscopy is the gold standard for diagnosis as it visualizes the paradoxical motion of the larynx and identifies precisely the site of obstruction, i.e., supraglottic, glottic, or both as in this report where closure of the anterior two-thirds of the glottis was associated with a small posterior chink during inspiration. Continuous laryngoscopy throughout an exercise test (2, 13) has been described in centers where specialist resources are readily available but may be difficult to implement in more conventional pediatric lung function laboratories. The inspiratory spirometry has been reported as a potential tool to identify EILO with characteristic flattening of the inspiratory loop. A recent review, however, showed the low diagnostic power of this feature (14).

The prolonged intra-breath description of Rrs was found useful in estimating the respective contribution of EILO and asthma from the following features. The response to exercise was dissociated in inspiration and expiration, Rrs exhibiting several bouts of dramatic increase in inspiration, while there was comparatively minor change in expiration. The pattern was clearly at variance with that usually observed in induced bronchoconstriction, where Rrs increases in a stable and sustained manner, in both inspiration and expiration. It is noteworthy that severe bronchoconstriction may trigger laryngeal mechanisms that protect from expiratory flow limitation, but these consist in reflex glottis closure in expiration rather than inspiration (15). Moreover, the deep breath after exercise was found to trigger a marked Rrs increase in inspiration. In contrast, when moderate exercise-induced bronchial obstruction is only present, a transient reduction of Rrs occurs after the deep breath, of similar magnitude in inspiration and expiration (16).

The current measurements of lung function were also found helpful in identifying inducers and facilitators of laryngeal dysfunction. While exercise was the major trigger, the laryngeal obstruction could be clearly re-induced in the post-exercise period with even minor changes in the breathing pattern. For instance, after alteration in the tidal expiratory flow suggestive of swallowing (Figure 3), the laryngeal obstruction was reinitiated. Also, there were indications throughout the post-exercise period that deep inhalation would re-induce intermittent laryngeal closure in inspiration (Figure 4). Such mechanism occurring during a spirometry maneuver would have contributed to limit the inhaled volume, hence FVC and FEV1. In fact the mild reduction in FEV1 observed 5 min after exercise was associated with a proportional reduction in FVC—and no change in FEV1/FVC—a case that may well have resulted from a sub-maximal inspiration induced by the laryngeal dysfunction. Altogether, the only firm indicator for asthma was the reversibility of FEV1 and FEV1/FVC after salbutamol.

It is concluded that examining EILO and asthma by the timely coordination of videolaryngoscopy and intra-breath analysis of Rrs throughout a standardized provocation protocol may allow a better characterization of anatomo-functional correlates of the laryngeal breathing disorder and the respective role of upper and lower airways. Moreover, inducers or facilitators such as deep breaths may be identified non-invasively. Further studies are needed to assess the specificity and sensitivity of Rrs in identifying EILO especially in asthmatic subjects.

## Ethics Statement

This study was carried out in accordance with the recommendations of Ethics Committee CPP Est III with written informed consent from the subject and his parents. The subject and his parents gave written informed consent in accordance with the Declaration of Helsinki.

## Author Contributions

AB, SB, LC, SD-A, CS, FM, and II prepared the project of this study. AB, SB, and LC performed videolaryngoscopy. LC-M, SM, and CB performed lung function tests, assured technical assistance during exercise challenge, and performed data collection. AB, SB, II, SD-A, LC, CS, and FM prepared the draft of manuscript. All the authors revised the final manuscript.

## Conflict of Interest Statement

The authors declare that the research was conducted in the absence of any commercial or financial relationships that could be construed as a potential conflict of interest.
